# Greatest changes in objective sleep architecture during COVID-19 lockdown in night owls with increased REM sleep

**DOI:** 10.1093/sleep/zsab075

**Published:** 2021-03-26

**Authors:** Jean-Louis Pépin, Sébastien Bailly, Ernest Mordret, Jonathan Gaucher, Renaud Tamisier, Raoua Ben Messaoud, Pierrick J Arnal, Emmanuel Mignot

**Affiliations:** 1 HP2 Laboratory, INSERM U1042, University Grenoble Alpes, Grenoble, France; 2 EFCR Laboratory, Grenoble Alpes University Hospital, Grenoble, France; 3 Dreem SAS, Science Team, Paris, France; 4 Center for Sleep Sciences and Medicine, Stanford University, Palo Alto, CA, USA

**Keywords:** sleep architecture, COVID-19 lockdown, chronotype, sleep-monitoring headband

## Abstract

**Study Objectives:**

The COVID-19 pandemic has had dramatic effects on society and people’s daily habits. In this observational study, we recorded objective data on sleep macro- and microarchitecture repeatedly over several nights before and during the COVID-19 government-imposed lockdown. The main objective was to evaluate changes in patterns of sleep duration and architecture during home confinement using the pre-confinement period as a control.

**Methods:**

Participants were regular users of a sleep-monitoring headband that records, stores, and automatically analyzes physiological data in real time, equivalent to polysomnography. We measured sleep onset duration, total sleep time, duration of sleep stages (N2, N3, and rapid eye movement [REM]), and sleep continuity. Via the user’s smartphone application, participants filled in questionnaires on how lockdown changed working hours, eating behavior, and daily life at home. They also filled in the Insomnia Severity Index, reduced Morningness–Eveningness Questionnaire, and Hospital Anxiety and Depression Scale questionnaires, allowing us to create selected subgroups.

**Results:**

The 599 participants were mainly men (71%) of median age 47 (interquartile range: 36–59). Compared to before lockdown, during lockdown individuals slept more overall (mean +3·83 min; *SD*: ±1.3), had less deep sleep (N3), more light sleep (N2), and longer REM sleep (mean +3·74 min; *SD*: ±0.8). They exhibited less weekend-specific changes, suggesting less sleep restriction during the week. Changes were most pronounced in individuals reporting eveningness preferences, suggesting relative sleep deprivation in this population and exacerbated sensitivity to societal changes.

**Conclusion:**

This unique dataset should help us understand the effects of lockdown on sleep architecture and on our health.

**Statement of Significance**

Objective sleep data were collected repeatedly over several weeks before and after COVID-19 lockdown, at home using a sleep-monitoring headband. Overall, during lockdown, individuals slept more, had less deep sleep, more light sleep, and longer rapid eye movement (REM) sleep (when one dreams). During lockdown, participants exhibited less weekend-specific changes in their sleep patterns than before lockdown. The strongest effects were in “night owls” with notably longer REM sleep. This was a unique opportunity to confirm or challenge previously published subjective data. It should help us to better understand the effects of lockdown on our health and productivity, particularly in some specific subgroups.

## Introduction

Nationwide home confinement has been extensively used across the world to prevent the spread of COVID-19 infection [1–3]. More than a billion people worldwide, a quarter of the world’s total population, have been confined to their homes. Stress, deterioration in mental health, anxiety, and depression have been reported as consequences. This, added to the fact that worldwide hundreds of thousands of deaths have occurred, is leading to an unprecedented level of ongoing stress [3, 4].

In contrast to mental health consequences, the effects of home confinement on sleep have not been well characterized, with studies only analyzing subjective reports collected by questionnaires or online surveys [5–7]. Yet, sleep has the potential to be affected by multiple factors: psychological effects secondary to financial difficulties [8], restriction of social activities [4], loss of external synchronizers such as social activities and regular meal and sleep times, and reduction in physical activity [9]. This, together with documented exacerbations of preexisting familial and couple conflicts as well as increased alcohol consumption and addiction [10], suggests that home confinement might also have devastating effects on sleep.

**Figure 1.**
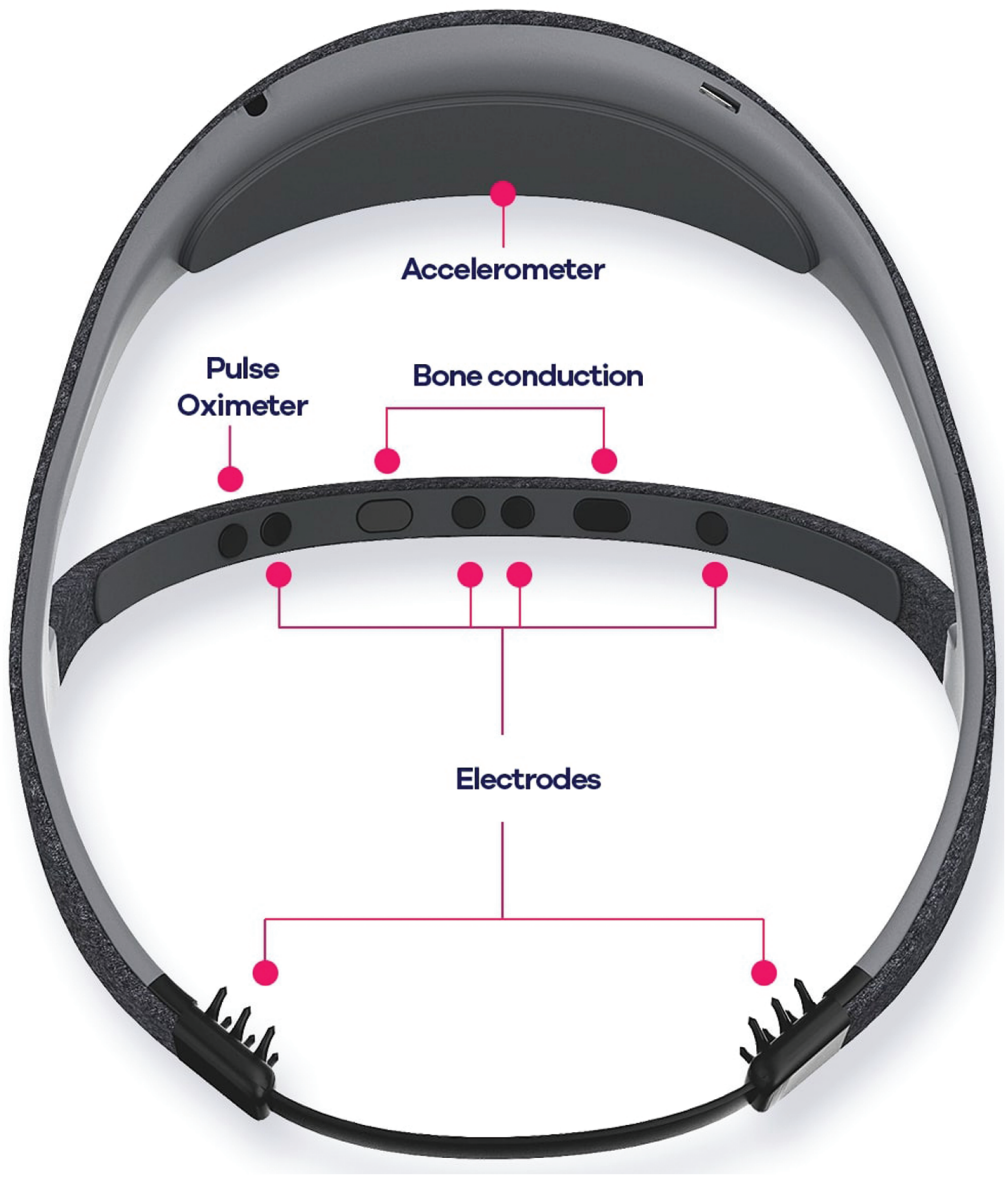
The Dreem headband device allowing to repeat sleep studies at home in ecological conditions.

The aim of this study was to assess the impact of home confinement on objective sleep parameters collected repeatedly during several nights over a 5-week period at home, before (reference period) and again during lockdown, in combination with subjective assessments. To record sleep, we used the Dreem headband (DH) [11] (Figure 1), a user-friendly, validated alternative to polysomnography (PSG) that allows sleep staging with accuracies similar to those of experienced scorers [12]. Unlike PSG, which is performed in a sleep clinic, the Dreem headband can be used every night in the user’s home to make repeated overnight measurements, and this, in his/her usual bed.

As some individuals are at a higher risk of developing sleep problems than others, a secondary objective was to analyze these modifications in subgroup populations with preexisting sleep complaints or with different chronotypes.

## Methods

### Study population

Regular users of the Dreem sleep-monitoring headband residing in France were contacted for voluntary participation. These are principally middle-aged active workers, of generally high socioeconomic class, mostly men wanting to monitor their sleep on a regular basis to improve well-being and diurnal performances (Table 1). All included individuals provided informed consent for the use of their anonymized data for research purposes.

**Table 1.** Population description and lockdown environment (*N* = 599)

	Median (IQR) or *N* (%)
*Study population characteristics*	
Age	47 (36–59)
Sex	
Male	424 (71)
Female	175 (29)
Chronotype (*N* = 424)	
rMEQ score	15 (12–17)
Morningness	97 (23)
Eveningness	78 (18)
Neither	249 (59)
Moderate to severe symptoms of anxiety (HADS-A >10)	112 (19)
Moderate to severe symptoms of insomnia (ISI ≥10)	366 (61)
*Patient’s ecosystem during lockdown*	
Sharing home with baby, young children, or teenager	168 (29)
Surrounding sleep environment compared to before lockdown:	
Quieter	266 (45)
As noisy	311 (53)
Noisier	15 (3)
Usage of screens and digital media in the 2 h preceding bedtime:	
Less screen use than before lockdown	32 (5)
As much screen use than before lockdown	419 (71)
More screen use than before lockdown	143 (24)
On furlough	175 (29)

rMEQ = reduced Morningness–Eveningness Questionnaire; ISI = Insomnia Severity Index; HADS-A = Hospital Anxiety and Depression Scale—anxiety section.

### Assessments

#### Longitudinal assessment of objective sleep parameters using the sleep-monitoring headband.

The DH device (Dreem SAS, Paris; Figure 1) is a wireless headband worn during sleep which records, stores, and automatically analyzes physiological data in real time. Both Dreem 1 and Dreem 2 headbands were used in this study. Sleep is objectively measured by sensors embedded in the device including (1) five EEG dry electrodes yielding seven EEG derivations (FpZ-O1, FpZ-O2, FpZ-F7, F8-F7, F7-01, F8-O2, FpZ-F8; 250 Hz with a 0.4–35 Hz bandpass filter); (2) a 3-D accelerometer located over the head that measures movements, position, and breathing frequency; and (3) a red-infrared pulse oximeter located in the frontal band which measures heart rate. The DH device is made of foam and fabric and has an elasticated band behind the head that makes it adjustable such that it is tight enough to be secure, but loose enough to minimize discomfort. Additional details have been published elsewhere [11]. DH automatic sleep staging has shown an overall accuracy similar to the average of five sleep scoring experts. Using the sleep-monitoring headband, we were able to repeatedly measure sleep architecture nearly every night: sleep latency (also called sleep onset duration [SOD]), total sleep time (TST), and duration of respective sleep stages (N2, N3, and rapid eye movement [REM]). For sleep microarchitecture, we assessed the number of sleep stage transitions (continuity) and spindle density. A minimum number of five nights before and five nights after lockdown was required to be included in the analysis. The initial value in the pre-lockdown period was considered as the reference to compute shifts in sleep duration over time.

#### Subjective measurements.

The DH connects to a mobile device (e.g. smartphone and tablet) via Bluetooth and transfers raw sleep data. The interface also allows the sending of questionnaires and surveys to users. During the 2 weeks following lockdown initiation, participants received a questionnaire asking them to document whether and how lockdown changed working hours, eating behavior, and daily life at home (working hours, screen and digital media usage, etc.). Volunteers were also asked about their sleep schedules prior to and during lockdown and to file-in insomnia (*Insomnia Severity Index* [ISI]), chronotype (reduced Morningness–Eveningness Questionnaire [rMEQ]), and anxiety (Hospital Anxiety and Depression Scale—anxiety [HADS-A]) questionnaires via the Dreem user’s smartphone application. The questionnaires were completed during the lockdown period and anonymized.

#### The Insomnia Severity Index.

The ISI is a seven-item scale that is scored using a 5-point Likert scale. It is a well-recognized and validated instrument used to assess insomnia [13, 14]. ISI scores range from 0 to 28, with total scores interpreted as follows: absence of insomnia (0–7), subthreshold insomnia (8–14), moderate insomnia (15–21), and severe insomnia (22–28). As typically done in other studies, a threshold of 10 was used to categorize insomnia.

#### The rMEQ chronotype questionnaire.

The total score from the five-item rMEQ ranges from 4 to 26. A higher score indicates morningness (eveningness: <12; neither: 12–17; morningness: >17) [15].

#### The Hospital Anxiety and Depression Scale.

The HADS is used to evaluate symptoms of anxiety and depression [16]. Evaluation consists of two subscales (anxiety, HADS-A and depression, HADS-D), each of which has seven items that are scored on a 4-point Likert scale. Total scores range between 0 and 21, with higher scores indicating more severe symptoms of anxiety or depression. As typically done in previous studies, we used a threshold of 10 to categorize anxiety for our analysis.

### Statistical analysis

Data are reported as median and interquartile range (IQR; Q1–Q3) for quantitative values and as numbers and percentage for qualitative values. As all variables were not normally distributed, we decided to present descriptive statistics as median and IQRs for all variables. Nonparametric Mann–Whitney tests were used to compare changes before and during lockdown between groups, and paired samples Wilcoxon tests to compare data before and during lockdown. Generalized linear mixed models with interaction terms were used to assess the independent effects of weekday–weekend and lockdown period on models. Sensitivity analyses were performed comparing sleep schedules and architecture (bedtime, wake-up time, TST, N2 duration, N3 duration, REM duration, and sleep onset latency [SOD]) during pre-lockdown and lockdown periods according to the following subgroups of interest: (1) insomnia subgroups: ISI ≥10 vs ISI <10, (2) anxiety subgroups: HADS-A >10 vs HADS-A ≤10, (3) on furlough vs not, (4) increase in screen time vs not, and (5) morningness vs eveningness chronotypes.

We conducted a post-hoc analysis to document the number of REM sleep episodes before and during lockdown. Statistical analyses were performed by using SAS v9.4 (SAS Institute Inc., Cary, NC) and R v3.6.1 software (R Foundation for Statistical Computing, Vienna, Austria). A *p* value of less than 0.05 was considered as statistically significant.

## Results

### Study population and subjective evaluation

The 599 study participants were regular French users of the DH with at least 5 weeks of nightly monitoring before and after lockdown (March 17, 2020). They were mainly men (71%) with a median age of 47 years (36–59) and 28.6% shared home living during lockdown with a baby, young children, and/or teenagers. Responses to rMEQ showed that 23% and 18.4% exhibited a primary morningness or eveningness chronotype, respectively. Moderate to severe symptoms of anxiety (HADS-A >10/21) were self-reported by 18.7% of participants, and moderate to severe symptoms of insomnia (ISI ≥10/28) were reported by 61%. During lockdown, self-reported questionnaires revealed that (1) wake-up time was delayed in 48.7% of participants, (2) the surrounding sleep environment was quieter compared to before lockdown in 45% of cases, (3) 23.9% of the sample reported increased usage of screens and digital media in the 2 h preceding bedtime, and (4) 49% of participants reported more sleep disturbances during home confinement (Table 1).

### Changes in sleep schedules and sleep architecture during lockdown: objective assessment through longitudinal sleep studies conducted using the sleep-monitoring headband

#### Sleep schedules and duration

##### Overall study population.

**Figure 2.**
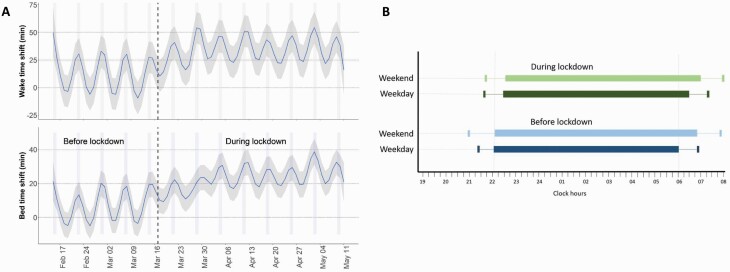
Sleep schedules before and during lockdown (whole study population). (A) Shifts in bedtime and wake-up times. The gray shaded area represents 95% confidence intervals. A positive shift (+) indicates later wake-up time in comparison to the initial value in the pre-lockdown period. By using a linear mixed model, we found a significant difference in sleep schedule when comparing week and weekend (*p* < 0.01) but no effect of lockdown (*p* = 0.54). (B) Actual bedtimes (i.e. lights out time) and wake-up times.

A progressive shift in bedtime (i.e. lights out time) and wake-up time was observed after 4–5 days of lockdown (Figure 2). On average, bedtime was delayed by 15 min (1–36) and lockdown had an even stronger effect on wake-up time, +21 min (1–48). There was an increased time in bed (TIB) of 8 min (−5 to 25; Wilcoxon paired test *p* < 0.01) and TST (*p* < 0.01). Before lockdown, sleep schedules and duration varied when comparing week and weekend nights (Figure 2 and Supplementary Table S1), *p* < 0.01. These week–weekend variations were no longer significant during lockdown, *p* = 0.54 (linear mixed model).

##### Sensitivity analysis in subgroups of interest.

**Figure 3.**
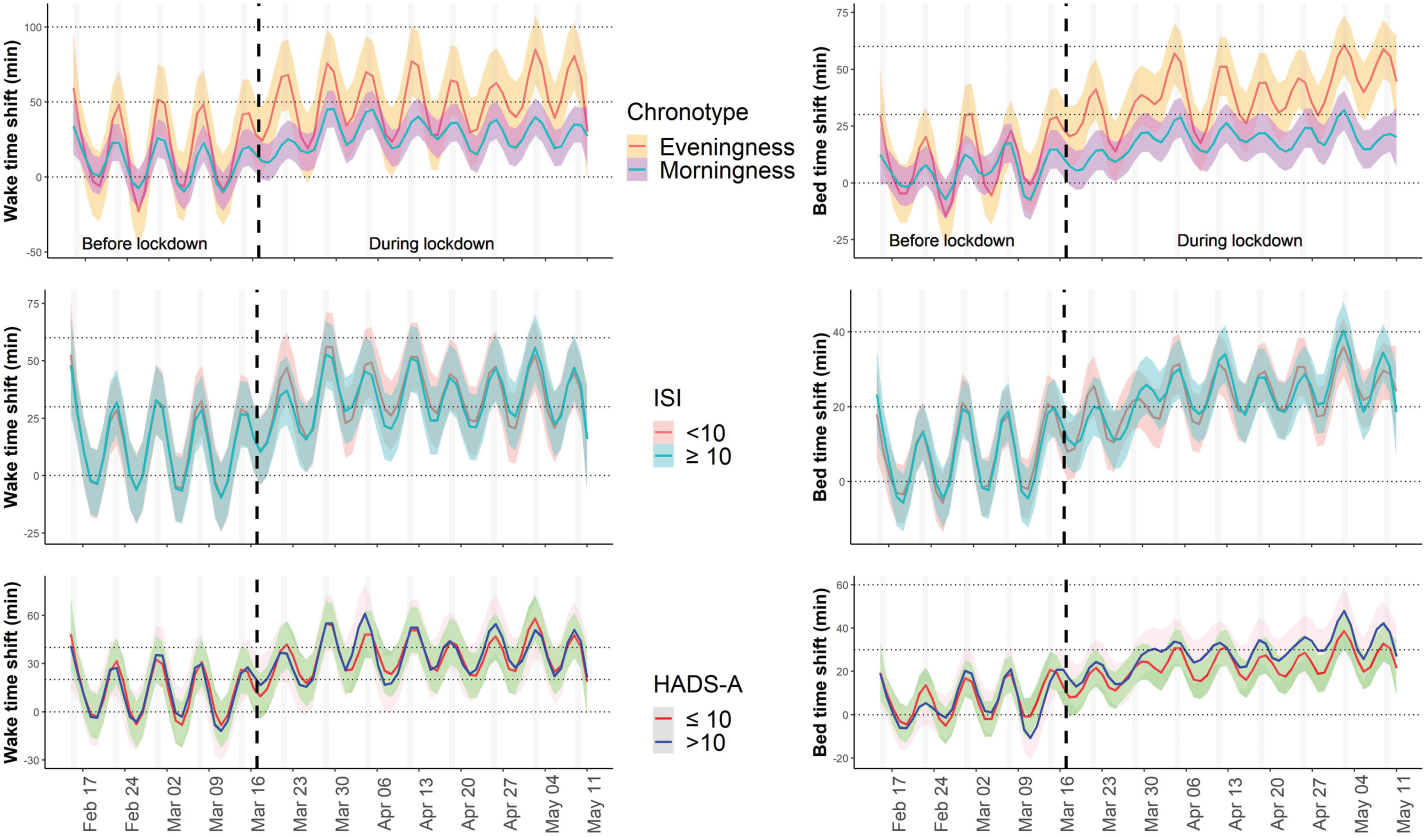
Sleep schedules in selected subpopulations. Bedtime and wake-up time before and during lockdown in morningness and eveningness chronotypes, insomnia (ISI <10 and ≥10), and anxiety (HADS-A ≤10 and >10) subpopulations. The gray shaded area represents 95% confidence intervals. A positive shift indicates later wake-up time in comparison to the initial value in the pre-lockdown period.

Analysis by chronotype subgroup indicated greater shifts in wake-up time and bedtime in eveningness compared to morningness chronotypes (Figure 3; *p* < 0.01).

#### Sleep architecture

**Figure 4.**
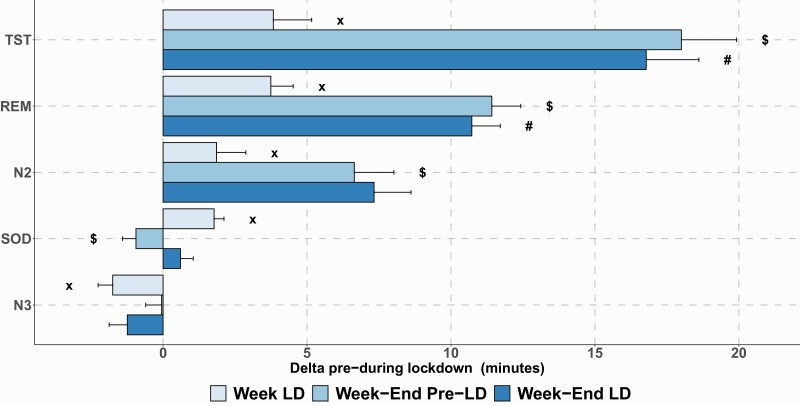
Changes in sleep architecture before and during lockdown (overall population) compared to pre-lockdown weekday nights (reference). TST = total sleep time; REM = rapid eye movement sleep; N2 = light sleep; SOD = sleep onset duration; N3 = deep sleep. x, significant lockdown effect; $, significant weekend effect; #, significant interaction between lockdown and weekend effects.

Pre- vs post-lockdown comparisons of objective sleep parameters such as SOD (also called sleep latency), sleep stages N2, and N3 of non-REM sleep and REM sleep are presented in Table 2 and Figure 4.

**Table 2.** Objective sleep parameters for whole study population

		Pre-lockdown		During lockdown	
		Weekdays	Weekend	Weekdays	Weekend
*Sleep duration and continuity*					
Total sleep time (min)		394 (361 to 425)	411 (368 to 453)	398 (363 to 431)	408 (364 to 453)
Delta TST (min)	Raw data*	Ref	16 (−9 to 46.6)	5 (−13 to 23)	14 (−10 to 43)
	Adjusted^†^		18 (±1.9)^‡^	3.8 (±1.3)^‡,§^	16.8 (±1.8)^‡,||^
Time in bed (min)		456 (423 to 489)	476 (439 to 514)	463 (429 to 495)	474 (432 to 518)
Delta TIB (min)	Raw data	Ref	17 (−8 to 51)	5 (−10 to 24)	14 (−9 to 45)
	Adjusted		20 (±2)^‡^	6 (±1.4)^‡^	−8 (±2)
Sleep efficiency (%)		88 (83 to 91)	88 (82 to 92)	87 (82 to 91)	88 (83 to 92)
Delta sleep efficiency (%)	Raw data	Ref	0.4 (−2 to 2)	−0.3 (−2.3 to 2)	0.1 (−2 to 3)
	Adjusted		−0.06 (±0.2)	−0.31 (±0.17)	0.52 (±0.25)
WASO (min)		19 (10 to 36)	20.3 (9 to 38)	20 (9 to 36)	19 (9 to 35)
Delta WASO	Raw data	Ref	0.2 (−6 to 7)	−0.2 (−6 to 5)	−0.6 (−8 to 7)
	Adjusted		1.3 (±0.7)	−0.14 (±0.6)	−1.1 (± 0.8)
*Sleep architecture*					
N2 (min)		194 (166 to 225)	202 (170 to 234)	196 (170 to 227)	203 (174 to 233)
Delta N2 (min)	Raw data	Ref	7 (−11 to 26)	1.9 (−12 to 17)	5.6 (−12 to 26)
	Adjusted		6.6 (±1.4)^‡^	2 (±1)^‡^	7 (±1.3)
N3 (min)		80 (59 to 100)	81 (60 to 101)	80 (59 to 99)	81 (59 to 99)
Delta N3 (min)	Raw data	Ref	−0.3 (−8 to 7)	−1.5 (−9 to 5)	−1.5 (−10 to 8)
	Adjusted		−0.05 (±0.55)	−1.75 (±0.51)^‡^	−1.24 (±0.63)
REM (min)		113 (88 to 134)	124 (98 to 148)	115 (95 to 138)	120 (98 to 147)
Delta REM (min)	Raw data	Ref	10 (−3 to 23)	4 (−7 to 15)	10 (−4 to 25)
	Adjusted		3.7 (±0.8)^‡^	11.4 (±1)^‡^	10.7 (±1)^‡^
SOD (min)		19 (13 to 27)	17 (12 to 24)	20 (14 to 28)	18. (13 to 26)
Delta SOD (min)	Raw data	Ref	−1.4 (−6 to 2)	1.2 (−2.3 to 6)	−0.1 (−4 to 5)
	Adjusted		−0.9 (±0.5)^‡^	1.8 (±0.3)^‡^	0.6 (±0.4)

WASO = wake time after sleep onset; SOD = sleep onset delay/latency (time from lights out to falling asleep).

*Raw data: data are presented as the median difference and interquartile range with weekday pre-lockdown values as reference.

^†^Adjusted: estimation of the mixed model with a random effect for the user, adjusted on age and gender (standard error).

^‡^Significant effect of the lockdown or interaction between weekend and lockdown compared to weekday pre-lockdown (reference).

^§^This corresponds to the lockdown effect.

^||^This corresponds to the interaction term between weekend and lockdown and can be interpreted as the cumulative effect of the weekend during lockdown compared to the week pre-lockdown.

##### Overall study population.

During lockdown, there was a significant increase in N2 (*p* = 0.03), REM (*p* < 0.01), and in latency to go to sleep (SOD; *p* < 0.01) by comparison with weeknights before lockdown. There was a significant decrease in N3 (*p* < 0.01). There was no difference in the number of REM sleep episodes before and during lockdown either in the overall population or in subgroup analyses (Supplementary Table S2). We found no significant change in wake time after sleep onset and sleep efficiency.

##### Sensitivity analysis of objective sleep data in subgroups of interest.

**Figure 5.**
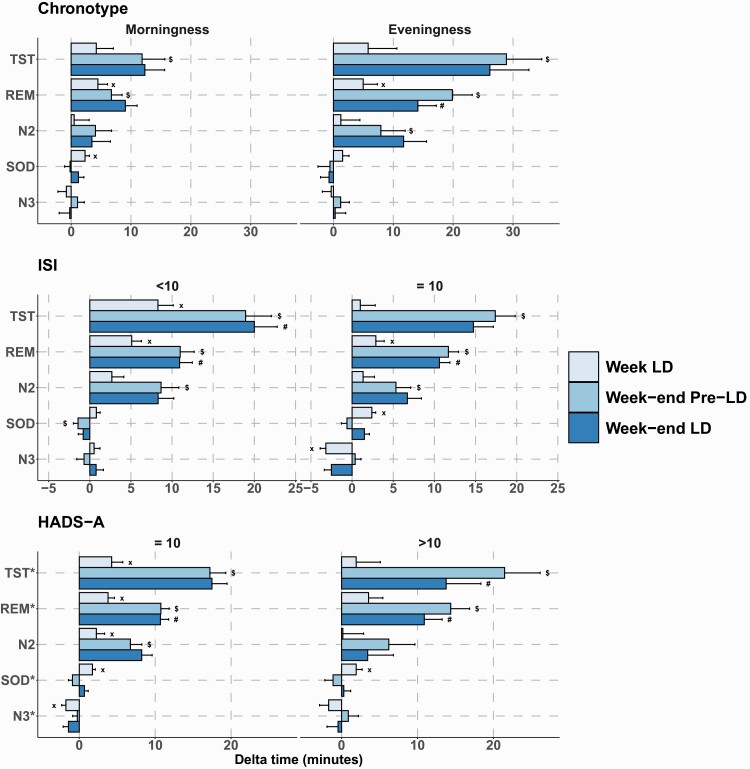
Changes in sleep architecture in selected subpopulations. Changes in sleep architecture before and during lockdown compared to pre-lockdown weekday nights (reference) in (A) morningness and eveningness chronotypes, (B) insomnia (ISI <10 and ≥10), and (C) anxiety (HADS-A ≤10 and >10) subpopulations. TST = total sleep time; REM = rapid eye movement sleep; N2 = light sleep; SOD = sleep onset duration; N3 = deep sleep. x, significant lockdown effect; $, significant weekend effect; #, significant interaction between lockdown and weekend effects.

Figure 5 and Table 3 report changes in objective sleep parameters in the subpopulations of interest. Although we found no significant differences in sleep microarchitecture when comparing the two chronotypes, the eveningness chronotype had a significant increase in REM sleep (*p* < 0.01) and SOD (*p* < 0.01) between pre-lockdown and lockdown periods. For the insomnia subgroups, there were significant differences when comparing those with ISI ≥10 vs ISI <10. Specifically, increases in TST (*p* < 0.01), REM (*p* = 0.03), SOD (*p* < 0.01), and deep sleep (N3) (*p* < 0.01) were significantly greater in the subgroup with an ISI <10 compared to those with ISI ≥10. For the anxiety (HADS-A < or ≥10) subgroups, we found no significant difference comparing subgroups. The subgroups with the lowest HADS-A score had a significant increase in TST (*p* < 0.01), REM (*p* < 0.01), N2 (*p* = 0.03), and SOD (*p* < 0.01) time and a significant decrease in N3 time (*p* < 0.01) due to lockdown (pre-lockdown vs lockdown periods). Conversely, a significant increase in SOD time (*p* = 0.01) was only observed in the highest HADS-A (most anxious) subgroup.

**Table 3.** Comparison of lockdown and weekend effects on objective sleep architecture for selected subgroups

		Lockdown effect		Weekend effect		Interaction term		Between subgroup effect
		Min (*SD*)	*p*	Min (*SD*)	*p*	Min (*SD*)	*p*	*p*
Morningness chronotype, *N* = 97	TST	4.2 (2.85)	0.14	11.86 (3.81)	<0.01	12.32 (3.3)	0.30	TST: 0.95 REM: 0.29 N2: 0.41 SOD: 0.70 N3: 0.81
	REM	4.47 (1.61)	<0.01	6.73 (1.79)	<0.01	9.06 (1.96)	0.29	
	N2	0.5 (2.51)	0.84	4.07 (2.69)	0.16	3.47 (3.09)	0.76	
	SOD	2.32 (0.74)	<0.01	−0.19 (0.9)	0.79	1.21 (0.92)	0.38	
	N3	−0.8 (1.41)	0.57	1.07 (1.12)	0.24	−0.24 (1.77)	0.54	
Eveningness chronotype, *N* = 78	TST	5.79 (4.81)	0.23	28.89 (5.91)	<0.01	26.12 (6.53)	0.18	
	REM	4.97 (2.37)	0.03	19.88 (3.31)	<0.01	14.09 (3.09)	<0.01	
	N2	1.23 (3.18)	0.70	7.92 (4.06)	0.05	11.71 (3.83)	0.57	
	SOD	1.53 (1.07)	0.15	−0.58 (2.01)	0.77	−0.74 (1.41)	0.46	
	N3	−0.38 (1.49)	0.80	1.2 (1.46)	0.41	0.29 (1.75)	0.78	
ISI <10, *N* = 233	TST	8.29 (1.85)	<0.01	18.94 (3.13)	<0.01	20 (2.78)	0.02	TST: <0.01 REM: 0.03 N2: 0.77 SOD: <0.01 N3: <0.01
	REM	5.09 (1.19)	<0.01	10.99 (1.7)	<0.01	10.91 (1.55)	<0.01	
	N2	2.68 (1.46)	0.06	8.65 (2.16)	<0.01	8.3 (1.9)	0.20	
	SOD	0.8 (0.46)	0.08	−1.43 (0.56)	0.01	−0.79 (0.6)	0.89	
	N3	0.52 (0.72)	0.47	−0.7 (0.88)	0.58	0.76 (0.9)	0.34	
ISI ≥10, *N* = 366	TST	0.99 (1.82)	0.56	17.39 (2.42)	<0.01	14.74 (2.41)	0.17	
	REM	2.88 (1.02)	<0.01	11.7 (1.24)	<0.01	10.61 (1.28)	<0.01	
	N2	1.33 (1.39)	0.28	5.33 (1.78)	<0.01	6.71 (1.72)	0.93	
	SOD	2.39 (0.48)	<0.01	−0.62 (0.68)	0.34	1.49 (0.61)	0.61	
	N3	−3.19 (0.69)	<0.01	0.37 (0.71)	0.56	−2.52 (0.85)	0.63	
HADS-A ≤10, *N* = 487	TST	4.27 (1.47)	<0.01	17.21 (2.09)	<0.01	17.48 (1.99)	0.08	TST: 0.99 REM: 0.17 N2: 0.11 SOD: 0.13 N3: 0.40
	REM	3.79 (0.85)	<0.01	10.74 (1.1)	<0.01	10.69 (1.09)	<0.01	
	N2	2.25 (1.09)	0.03	6.74 (1.5)	<0.01	8.23 (1.37)	0.78	
	SOD	1.74 (0.38)	<0.01	−0.9 (0.52)	0.10	0.68 (0.5)	0.70	
	N3	−1.76 (0.56)	<0.01	−0.26 (0.61)	0.89	−1.42 (0.7)	0.42	
HADS-A >10, *N* = 112	TST	1.93 (3.17)	0.54	21.47 (4.69)	<0.01	13.77 (4.57)	0.03	
	REM	3.55 (1.88)	0.06	14.37 (2.49)	<0.01	10.89 (2.35)	<0.01	
	N2	0.16 (2.74)	0.95	6.21 (3.43)	0.06	3.43 (3.39)	0.37	
	SOD	1.92 (0.79)	0.01	−1.13 (1.08)	0.22	0.28 (0.91)	0.72	
	N3	−1.71 (1.2)	0.15	0.88 (1.33)	0.50	−0.47 (1.45)	0.68	

TST = total sleep time; SOD = sleep onset delay/latency (time from lights out to falling asleep); REM = rapid eye movement sleep; N2 = light sleep; N3 = deep sleep; ISI = Insomnia Severity Index; HADS-A = Hospital Anxiety and Depression Scale—anxiety; SD = standard deviation.

We conducted subgroup analyses for screen usage, on furlough or working from home, and living alone or with others (Supplementary Table S3 and Supplementary Figure S1, A–C). We found no significant impact of any of these subgroups on objectively measured sleep architecture during lockdown.

##### Sleep continuity and microarchitecture.

There was a significant reduction in the number of sleep stage changes/TST ratio (*p* < 0.01) during lockdown but no significant change in spindle density.

##### Sensitivity analysis of self-reported sleep data from questionnaires.

Variations in bedtime and wake-up time were nearly threefold higher in eveningness chronotypes (*p* < 0.01 for both bedtime and wake-up time) and were significantly higher in the subgroup who were on furlough (*p* = 0.01 and *p* < 0.01). Increase in sleep latency and reduction in N3 were significantly more marked in people with insomnia (ISI scores >10; *p* < 0.01).

## Discussion

This study provides new information on how objective sleep parameters changed during the COVID-19 lockdown. The current data set includes multiple longitudinal sleep studies repeated many nights in nearly 600 participants before and during lockdown. Across all participants, bedtime and wake-up time schedules were delayed and differences in the timing of sleep between weekdays and weekends were attenuated. Changes in sleep schedules appeared early after total national lockdown, suggesting rapid adjustments in individual home sleep routines in response to this dramatic societal change. We also found that sleep latency was prolonged, an effect associated with decreased slow-wave sleep (N3) and an increase in light (N2) and REM sleep. Importantly, changes in sleep schedules and objective sleep patterns were more pronounced in eveningness chronotypes or individuals with preexisting sleep problems.

Previous studies that have addressed the impact of COVID-19 lockdown on sleep have all been conducted using subjective questionnaires, online surveys [4–7, 17], or crowdsourced databases from smartphone applications [18]. These data consistently report that sleep-wake schedules markedly changed, with a later sleep onset in the evening and a later awakening in the morning. This was generally associated with an increase in TIB and self-reported impairment in sleep quality [19] and a higher use of hypnotics [20]. The main limitations of these studies were (1) a cross-sectional design without documentation of pre-lockdown sleep quality and (2) sleep changes were all self-reported, subjective by nature, and probably strongly influenced by the very particular and stressful context of the COVID-19 pandemic.

The major strength of our study is to provide longitudinal data on repeated sleep studies of the same individual. This allowed for the comparison of objective sleep parameters before and during lockdown. Using this design, we confirmed that sleep onset in the evening and awakening in the morning were delayed with median objective delays of 15 and 21 min, respectively. We also demonstrated a strong attenuation in sleep schedule differences between workdays and weekend days. Individuals normally change sleep schedules during weekends owing to weekend-related social activities and make adjustments in sleep duration to recover from sleep loss incurred during the working week [21]. During imposed isolation, the reduction in social exchanges and transferring work activities to the home are likely to have reduced these weekdays–weekends variations.

Some individuals partially correct chronic sleep deprivation with a return to their personal sleep needs, including sleep schedules more aligned with their own chronotype [22, 23]. A similar reduction in weekday–weekend variability has also been reported for physical activity measurements using wearable activity trackers [9]. Such variations in physical activity are certainly involved in the observed changes in sleep patterns.

Objective headband data did not confirm a previously reported large increase in TST [19], but an objective median increase in sleep duration of only around 20 min (~5% of TST) was observed.

The most striking change in sleep architecture we found was an increase in REM sleep duration. The increase in REM duration is essentially significant after adjustment for age and sex. As expected, the effect is particularly obvious in the subpopulation with the biggest changes in sleep schedules (i.e. evening chronotype). The physiological distribution of REM sleep across the night has a well-known preferential distribution toward the latter portion of the night, a phenomenon principally governed by the circadian clock [24–26]. As the sleep period was prolonged during lockdown, people generally reported going to sleep later and waking up later and as a consequence had increased REM sleep in the morning. This could explain reports in the media of more dreams during lockdown, although these have not been scientifically documented.

The magnitude of objective sleep parameter changes was expected to be more prominent in people with higher levels of anxiety, with more sleep complaints, or with an evening chronotype [22, 23, 27]. In agreement with this hypothesis, we found that variations in bedtime and wake-up time were greatest in eveningness chronotypes and in individuals reporting no work activities at home. This suggests that evening chronotypes were more sleep deprived at baseline (notably of REM sleep) due to difficulties in adapting to a societal work schedule that favors morning chronotypes. Interestingly, eveningness is a consistent risk factor for a host of mental and physical disorders and mortality [28]; and increased all-cause mortality has recently been reported in participants exhibiting less REM sleep during in-lab sleep studies in three separate cohorts [29, 30].

More surprisingly perhaps, the effects of the lockdown such as increased sleep duration were not significant in people with insomnia or anxiety (two frequently coexisting pathologies with likely shared causality [31]), although increases in sleep latency and reductions in N3 were strongest in people with ISI scores above 10 (Table 3). The smaller effect on objective sleep in this population may suggest that participants with anxiety and depression were already more sleep-satiated, i.e. are trying too hard to sleep, an important component of insomnia. As a consequence, increased available time for sleep due to lockdown may have had less effect on objective sleep architecture in this population. Importantly, however, objective sleep and subjective perception correlate poorly in this population. It is thus possible that the small changes in objective sleep seen in this population have strong subjective effects, explaining why other studies have described higher impacts of insomnia and more systematic use of hypnotics following lockdown [20, 32]. Similarly, analyzing fatigue in participants with insomnia, we found that this symptom was more pronounced in patients spending more time in bed [33], suggesting that attempting to sleep more may result in more subjective suffering. This would also be in line with the well-known efficacy of cognitive-behavioral therapy, which instructs patients to spend less time in bed [34]. Further studies are needed to assess if changes in the opportunity to sleep following lockdown mediate or exacerbate previously reported changes in mental health, or vice versa.

Our study has obvious strengths but also limitations. First, the study population is not representative of the general population. Regular DH users are probably more engaged than the general population in monitoring and improving their personal sleep health behaviors. However, the study sample has been well-documented regarding chronotype, sleep profile, and lockdown environment, allowing a more definitive characterization of specific responses when subjected to lockdown. More than 90% of participants were using the device’s deep sleep and sleep onset stimulation options with only marginal changes in patterns of usage before and during lockdown.

Self-reported questionnaires were sent out in a time window of 2 weeks after lockdown started. This might have induced a recall bias, but of limited importance owing to the short delay. The questionnaires were conducted during lockdown and informed on the respondent’s self-perception during this period but not on the variation of the severity of anxiety and insomnia between pre-lockdown and lockdown periods. Additionally, we did not have reliable data regarding the impacts of sleep apnea in the study population. Second, recordings using the DH are not equivalent to a full in-laboratory PSG. Nevertheless, the robustness of sleep staging has been validated and is considered equivalent to that of experienced manual scorers [12]. Also, the ability to repeatedly characterize sleep at home, during the week and at weekends, in what can be considered more “ecological conditions” is unique and may have more face validity than in-laboratory PSG results.

In conclusion, we have documented the impact of COVID-19 lockdown on objectively assessed sleep schedules and architecture for the first time, showing that its impact is more significant (and likely beneficial) in participants with an eveningness chronotype, and less pronounced in participants with insomnia or anxiety during lockdown. A logical next step may be to document the evolution of these changes and delay to restoration of pre-lockdown patterns once the strict lockdown period has ended, evaluating also ISI and HADS-A following return to normal behavior. These results also indicate strong heterogeneity in the effect of the lockdown in different subpopulations.

## Supplementary Material

zsab075_suppl_Supplementary_Material

## Data Availability

Deidentified participant data collected for the study, including individual participant data and a data dictionary defining each field in the set, will be made available for academic use on reasonable request to the corresponding author following publication through a signed data access agreement.
