# Supplementary material for: Greatest changes in objective sleep architecture during COVID-19 lockdown in night owls with increased REM sleep
Source: Sleep. 2021 Mar 26;44(9):zsab075. doi: 10.1093/sleep/zsab075 (PMC8083638; doi:10.1093/sleep/zsab075)
Supplement: zsab075_suppl_Supplementary_Material [file zsab075_suppl_supplementary_material.docx]

**Greatest changes in objective sleep architecture during COVID-19 lockdown in night-owls with increased REM sleep**

Jean-Louis Pépin^1,2,*^, Sébastien Bailly^1,2,*^, Ernest Mordret^3^, Jonathan Gaucher^1,2^, Renaud Tamisier^1,2^, Raoua Ben Messaoud^1,2^, Pierrick J. Arnal^3,ᴪ^ and Emmanuel Mignot^4,ᴪ^

^1^HP2 laboratory, INSERM U1042, University Grenoble Alpes, Grenoble, 38000 France,

^2^EFCR Laboratory, Grenoble Alpes University Hospital, Grenoble, 38043 France,

^3^Dreem SAS, Science Team, 124 Rue Réaumur, Paris, 75002 France,

^4^Center for Sleep Sciences and Medicine, Stanford University, Palo Alto, CA,94304 USA

*The two first authors have contributed equally to the manuscript

^ᴪ^Co-senior authors

**Corresponding author**

**Jean-Louis Pépin,** Laboratoire EFCR, CHU de Grenoble Alpes, CS10217, 38043, Grenoble, France. Email: [JPepin@chu-grenoble.fr](mailto:JPepin@chu-grenoble.fr)

**Supplementary Figure and Table Captions:**

**Table S1: Actual bedtime (i.e. lights out time) and wake up time**

**Table S2: Number of REM sleep episodes before and during lockdown**

**Table S3: Sub-group analyses for screen usage, on furlough or working from home, and living alone or with others**

**Figure S1 (A to C): Objective sleep parameters in subgroups of interest**

A-Screen usage

B-On furlough or Working from home

C-Living alone or with others

**Table S1: Actual bedtime (i.e. lights out time) and wake up time**

|  | **Bed time** | **Wake up time** |
| --- | --- | --- |
| Before lockdown |  |  |
| Weekday | 22:06 [21:20- 22:46] | 06:01 [05:20- 06:51] |
| Weekend | 22:07 [21:00- 22:55] | 06:51 [05:59- 07:50] |
| During lockdown |  |  |
| Weekday | 22:25 [21:43- 23:07] | 06:30 [05:47- 07:18] |
| Weekend | 22:34 [21:45 - 23:20] | 07:00 [06:02- 08:00] |

Values are presented as medians [interquartile range]

By using a linear mixed model, we found a significant difference in sleep schedule when comparing week and week end (p<0.01) but no effect of lockdown (p=0.54).

**Table S2: Number of REM sleep episodes before and during lockdown**

|  |  |  | Before lockdown | | Lockdown | |
| --- | --- | --- | --- | --- | --- | --- |
|  |  |  | Week | Weekend | Week | Weekend |
| REM (number of episodes) | All |  | 5.1 [4.4 ; 6.1] | 5.5 [4.75 ; 6.6] | 5.2 [4.5 ; 6.0] | 5.4 [4.5 ; 6.3] |
| Delta REM (number of episodes) |  | unadjusted | Ref | 0.4 [-0.2 ; 1.0] | 0.02 [-0.5 ; 0.5] | 0.2 [-0.4; 0.9] |
|  |  | adjusted |  | 0.4 (±0.04)* | 0.04 (±0.03) | -0.23 (±0.04)* |
| REM (number of episodes) | Chronotype  Morningness  N=97 |  | 5.1 [4.4 ; 6.0] | 5.6 [4.6 ; 6.4] | 5.2 [4.5 ; 6.1] | 5.3 [4.8 ; 6.0] |
| Delta REM (number of episodes) |  | unadjusted | Ref | 0.3 [-0.3 ; 0.9] | 0.03 [-0.4 ; 0.4] | 0.1 [-0.4; 0.6] |
|  |  | adjusted |  | 0.3 (±0.09)* | 0.1 (±0.09) | -0.26 (±0.1)* |
| REM (number of episodes) | Chronotype Eveningness  N=78 |  | 5.0 [4.2 ; 5.8] | 5.6 [4.9 ; 6.6] | 5.1 [4.5 ; 5.8] | 5.5 [4.5 ; 6.3] |
| Delta REM (number of episodes) |  | unadjusted | Ref | 0.6 [-0.07 ; 1.2] | 0.07 [-0.4 ; 0.6] | 0.4 [-0.2; 1.1] |
|  |  | adjusted |  | 0.7 (±0.12)* | 0.1 (±0.09) | -0.29 (±0.12)* |
| REM (number of episodes) | ISI < 10 |  | 5.1 [4.4 ; 6.1] | 5.6 [4.75 ; 6.6] | 5.2 [4.6 ; 6.0] | 5.5 [4.6 ; 6.3] |
| Delta REM (number of episodes) | N=233 | unadjusted | Ref | 0.3 [-0.3 ; 1.0] | 0.01 [-0.5 ; 0.5] | 0.2 [-0.4; 0.9] |
|  |  | adjusted |  | 0.5 (±0.07)* | 0.04 (±0.05) | -0.26 (±0.07)* |
| REM (number of episodes) | ISI ≥ 10 |  | 5.1 [4.4 ; 6.1] | 5.5 [4.7 ; 6.6] | 5.2 [4.4 ; 5.9] | 5.3 [4.5 ; 6.3] |
| Delta REM (number of episodes) | N=366 | unadjusted | Ref | 0.4 [-0.2 ; 1.0] | 0.03 [-0.5 ; 0.5] | 0.2 [-0.4; 0.9] |
|  |  | adjusted |  | 0.4 (±0.05)* | 0.04 (±0.04) | -0.21 (±0.06)* |

*significant effect of the lockdown or interaction between weekend and lockdown compared to weekdays pre-lockdown (reference). Adjustment for age and sex. Ref: Reference period.

**Table S3: Sub-group analyses for screen usage, on furlough or working from home, and living alone or with others**

|  |  | **Lockdown Effect** | | **Weekend Effect** | | **Interaction term** | | **Sub-group effect** |
| --- | --- | --- | --- | --- | --- | --- | --- | --- |
| Unchanged screen usage  N=456 | TST | 5 (1.5) | <.01 | 18.9 (2.2) | <.01 | 19.9 (2.1) | 0.07 | TST: 0.09  REM: 0.91  N2: 0.41  SOD: 0.87  N3: 0.06 |
|  | REM | 4.4 (0.9) | <.01 | 12 (1.2) | <.01 | 11.9 (1.1) | <.01 |  |
|  | N2 | 2.3 (1.2) | <.01 | 6.9 (1.6) | 0.04 | 9.0 (1.5) | 0.97 |  |
|  | SOD | 1.5 (0.74) | 0.04 | -1.0 (0.90) | <.01 | 0.4 (0.5) | 0.99 |  |
|  | N3 | -1.7 (0.6) | 0.73 | 0.1 (0.6) | <.01 | -1.0 (0.7) | 0.36 |  |
| Increased screen usage  N=143 | TST | 0.2 (2.9) | <.01 | 15.1 (3.9) | 0.93 | 6.9 (3.7) | 0.05 |  |
|  | REM | 1.6 (1.7) | <.01 | 9.7 (2.0) | 0.34 | 7.0 (2.1) | 0.04 |  |
|  | N2 | 0.4 (2.1) | 0.05 | 5.7 (2.8) | 0.82 | 1.9 (2.6) | 0.22 |  |
|  | SOD | 2.7 (0.6) | 0.70 | -0.3 (0.8) | <.01 | -1.2 (0.8) | 0.36 |  |
|  | N3 | -1.9 (1.0) | 0.88 | -0.4 (1.1) | 0.06 | -2.0 (1.2) | 0.97 |  |
| On furlough  N=429 | TST | 1.2 (1.6) | <.01 | 13.6 (2.2) | 0.44 | 12 (2.1) | 0.20 | TST: 0.28  REM: 0.40  N2: 0.57  SOD: 0.29  N3: 0.85 |
|  | REM | 2.0 (1.0) | <.01 | 9.4 (1.2) | 0.05 | 8.2 (1.2) | <.01 |  |
|  | N2 | 1.6 (1.3) | 0.01 | 4.1 (1.6) | 0.18 | 5.9 (1.5) | 0.80 |  |
|  | SOD | 1.6 (0.4) | 0.23 | -0.6 (0.6) | <.01 | 1.1 (0.5) | 0.89 |  |
|  | N3 | -2.3 (0.6) | 0.71 | 0.1 (0.7) | <.01 | -2.0 (0.8) | 0.77 |  |
| Working from home  N=170 | TST | 10.5 (2.3) | <.01 | 29.0 (3.5) | <.01 | 28.7 (3.5) | <.01 |  |
|  | REM | 8.1 (1.2) | <.01 | 16.5 (1.9) | <.01 | 17.1 (1.6) | <.01 |  |
|  | N2 | 2.6 (1.7) | <.01 | 12.9 (2.6) | 0.12 | 10.8 (2.3) | 0.12 |  |
|  | SOD | 2.2(0.6) | 0.05 | -1.7 (0.8) | <.01 | -0.7 (0.7) | 0.26 |  |
|  | N3 | -0.2 (0.9) | 0.77 | -0.4 (1.0) | 0.81 | 0.8 (1.1) | 0.23 |  |
| Living alone  N=347 | TST | 5.1 (1.8) | <.01 | 19.4 (2.4) | <.01 | 17.5 (2.3) | <.01 | TST: 0.16  REM: 0.84  N2: 0.40  SOD: 0.06  N3: 0.25 |
|  | REM | 3.4 (1.0) | <.01 | 11.2 (1.3) | <.01 | 10.3 (1.2) | <.01 |  |
|  | N2 | 2.5 (1.4) | <.01 | 8.1 (1.7) | 0.07 | 7.3 (1.7) | 0.11 |  |
|  | SOD | 1.2 (0.4) | 0.01 | -1.3 (0.5) | <.01 | -0.4 (0.5) | 0.65 |  |
|  | N3 | -0.7 (0.7) | 0.52 | -0.1 (0.7) | 0.26 | 0.0 (0.8) | 0.54 |  |
| Living with others  N=221 | TST | 1.6 (2.1) | <.01 | 14.3 (3.3) | 0.42 | 13.4 (3.2) | 0.52 |  |
|  | REM | 3.8 (1.3) | <.01 | 11.6 (1.7) | <.01 | 10.4 (1.8) | <.01 |  |
|  | N2 | 0.7 (1.7) | 0.21 | 3.2 (2.5) | 0.55 | 6.0 (2.2) | 0.42 |  |
|  | SOD | 2.6 (0.6) | 0.65 | -0.5 (0.9) | <.01 | 2.3 (0.9) | 0.90 |  |
|  | N3 | -2.9 (0.8) | 0.54 | -0.4 (0.9) | <.01 | -2.9 (1.0) | 0.53 |  |

**Figure S1 (A to C): Objective sleep parameters in subgroups of interest**

A-Screen usage

B-On furlough or Working from home

C-Living alone or with others

**A**

**
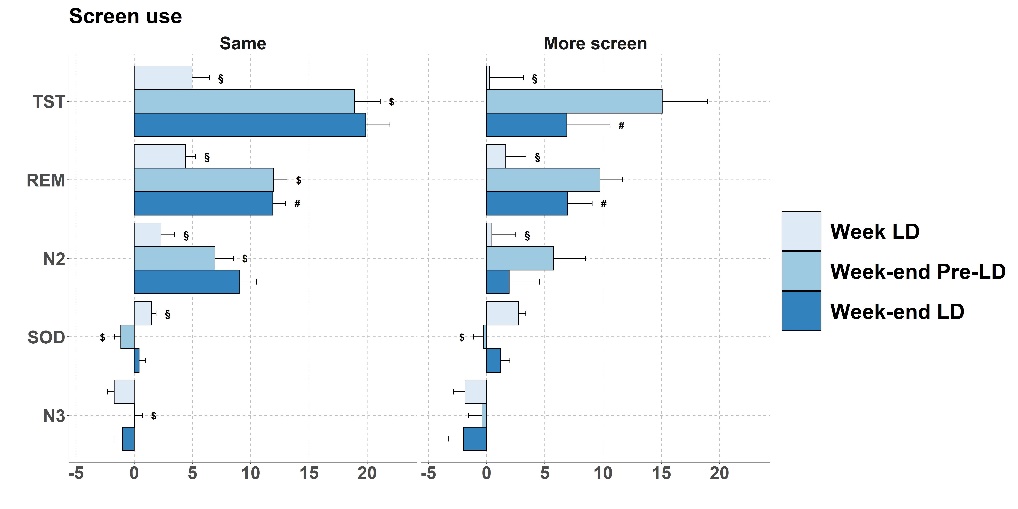
**

**B**

**
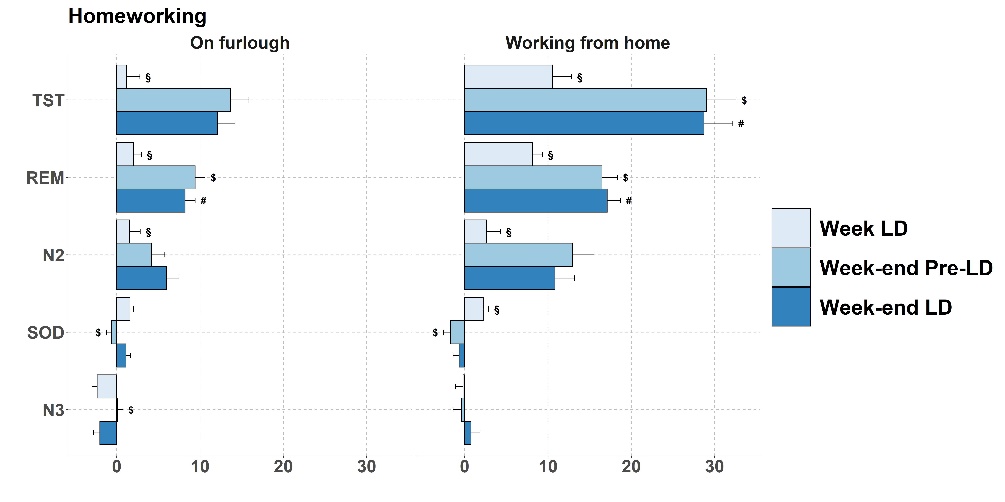
**

**C**

**
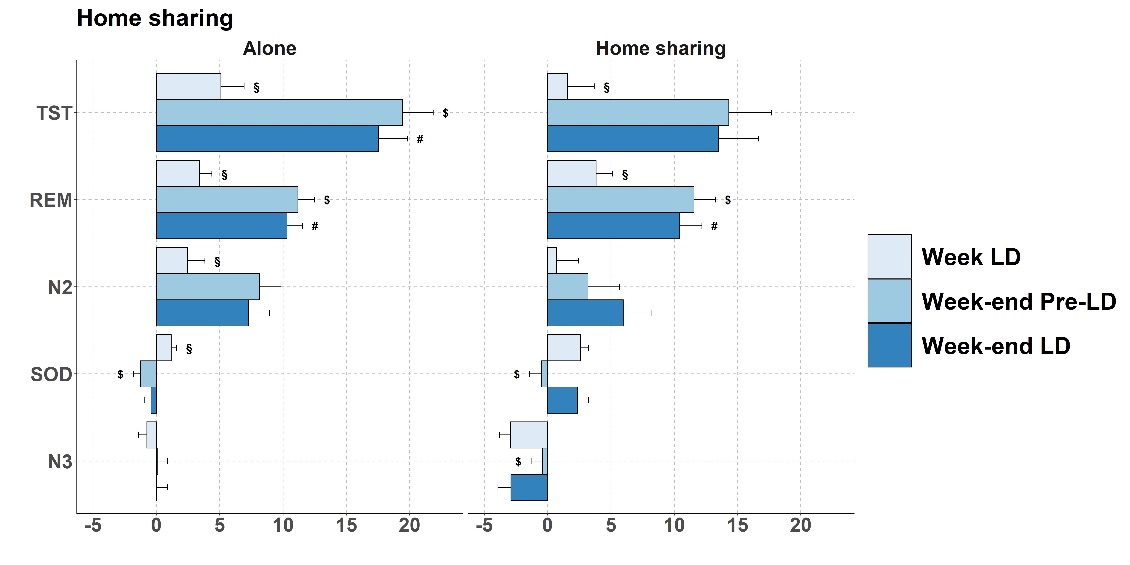
**
